# The Impact of Education and Insurance Status on Past-Year Dental Visits Among Older Mexican Adults: Results From the 2001 and 2012 Mexican Health and Aging Study

**DOI:** 10.1177/08982643221086586

**Published:** 2022-04-23

**Authors:** Jennifer Archuleta, Hiram Beltrán-Sánchez

**Affiliations:** 1 Fielding School of Public Health, 25808 University of Los Angeles, CA, USA

**Keywords:** oral health, Mexico, socioeconomic status, health policy

## Abstract

**Objective:** This study assessed past-year dental visits among older Mexican adults from the Mexican Health and Aging Study (MHAS). MHAS is a nationally representative cohort study of adults 50 years and older from Mexico. **Methods:** Baseline data from 2001 were compared with 2012 data. Binary logistic regression identified significant predictors of past-year dental visits. Decomposition techniques examined factors that contributed to changes in dental visits between 2001 and 2012. **Results:** Education and insurance status were positively associated with past-year dental visits, while decomposition results showed that population composition (more adults receiving insurance and higher education over time) contributed to the increased prevalence of dental visits between 2001 and 2012. **Discussion:** Education and insurance are critical factors that govern access to oral healthcare. After the provision of universal dental coverage by Mexico’s *Seguro Popular* in 2003, our results may reflect promising effects of such programs, which can inform future policies in Mexico and other settings.

## Introduction

Equitable access to dental care is necessary for supporting oral health throughout the life course. This is particularly important among older adults as their access to health care in general, and oral health in particular, is typically dependent on retirement benefits that may or may not include coverage for oral health (e.g., Medicare in the United States does not cover dental care). There are many health consequences derived from poor oral health. For example, poor oral health is the leading global cause of disability-adjusted life years among adults aged 65 and older (Marcenes et al., 2013). Oral diseases such as permanent tooth loss, untreated dental caries, and gum infections impact more than 77% of older adults worldwide (Kassebaum et al., 2017). Without proper treatment, oral diseases can increase pain and lead to difficulty with eating, speaking, and swallowing (Furuta & Yamashita, 2013; Naka et al., 2014). Good oral hygiene practices such as brushing, flossing, and routine dental visits are important for the detection and treatment of oral health issues (Coll et al., 2020).

Some evidence suggests that older adults are less likely to have access to dental care which in turn impacts their oral health. For example, an assessment of 194 countries showed that compared to younger age groups, older adults, especially those from low- to middle-income countries, were most affected by oral diseases and had the lowest access to dental care (Peres et al., 2019). Similar trends in Mexico illustrated that only about half of Social Security beneficiaries aged 60 and older had received any oral health service within the past year in the early 2000s (Sánchez-García et al., 2007). Moreover, among older Mexican adults, the likelihood of permanent tooth loss and dental caries increased with each additional year of age (Sánchez-García et al., 2014). In addition, poor oral health status was found to be associated with frailty and lower quality of life among Mexican adults over age 70 (Castrejón-Pérez et al., 2017; Ortíz-Barrios et al., 2019). Despite the need for consistent preventive and emergency dental health services within this population, evidence from the early 2000s suggested that many Mexican older adults lacked access to any type of dental care (Sánchez-García et al., 2007).

The use of oral health services is disproportionately impacted by inequities in socioeconomic status (SES). Studies in 23 countries including Brazil, China, Mexico, and the United States found that older adults with lower levels of income, educational attainment, and dental insurance were less likely to utilize dental care services and were simultaneously more likely to have worse oral health outcomes (Almeida et al., 2017; Andrade et al., 2020; Hernández-Palacios et al., 2015; Sánchez-García et al., 2007). Prior research suggests a positive relationship between high educational attainment and dental visitations among older adults. In many middle- to high-income countries such as the United States, the use of dental care by older adults was more frequent among individuals with higher levels of education than those with fewer years of education (Almeida et al., 2017; Andrade et al., 2020; Ramírez et al., 2011). Among Mexican older adults, access to dental care was also found to be associated with greater educational attainment (Almeida et al., 2017; Andrade et al., 2020).

Additional factors such as comorbidities, gender, and age are also known to influence differential access to dental care. Previous research among older adults from Germany has shown that dental visitations declined with rising age but at a faster rate among women (Spinler et al., 2019). In the United States, females above age 65 were more likely to have a dental visit compared to older males (Gironda et al., 2013; Marchini et al., 2020). Studies in Mexico also found that women aged 60 and older were more likely to utilize dental health services compared to older adult men (Sánchez-García et al., 2007).

Comorbidities in older age were found to have an inverse association with dental care utilization. Older U.S. adults with mobility limitations and chronic conditions such as diabetes, heart problems, and hypertension had low compliance with seeking dental care in the past 12 months (Luo et al., 2018; Ramírez et al., 2011). Furthermore, dental visits and services were found to be less frequent among U.S. older adults with depression and anxiety and those exhibiting symptoms of loneliness and low social support (Burr & Lee, 2013; Okoro et al., 2012). Symptoms of frailty such as exhaustion, weight loss, weakness, and low physical activity were associated with fewer dental visits and poorer self-rated oral health status among older adults in Mexico (Castrejón-Pérez et al., 2012). These trends suggest that comorbidities can discourage older adult populations from seeking oral health treatment due to additional healthcare costs, time constraints, and physical burdens.

In the case of Mexico, important health care policy changes implemented in the early 2000s can also have an impact on access to dental care among older adults. *Seguro Popular* is a public health insurance program that was enacted in Mexico beginning in 2002. This program expanded healthcare services to over 50 million individuals who previously did not receive formal sector healthcare insurance from employers, private insurance, or social security. Unlike U.S. Medicare, *Seguro Popular* offers access to seven different categories of oral healthcare services for older adults including emergency and preventive dental visits (Comisión Nacional de Protección Social en Salud, 2012; Gutiérrez, 2014). Some evidence suggests that *Seguro Popular* has increased overall healthcare utilization and the use of diagnostic tests among older adults (Parker et al., 2018).

Along with health insurance, educational attainment among older adults also steadily increased in Mexico between 2001 and 2012. On average, educational attainment was found to be higher among the 2012 wave of older adults than the wave that was interviewed a decade earlier (Díaz-Venegas et al., 2019). The difference in educational attainment between both waves of older adults is an important demographic shift. Changes in the population composition might influence the extent to which an increasingly educated population accounts for an increase in prevalence of past-year dental visits. For example, individuals with more schooling may be more aware of the importance of seeking dental health care and also more likely to utilize the newly created Seguro popular which may lead to a higher prevalence of past-year dental visits in 2012 relative to 2001. Thus, if the proportion of past-year dental visits is greater in 2012 than in 2001, we must discern how much of this change in the prevalence is driven by the population composition (i.e., a larger proportion of the population who attain higher schooling in 2012 vs. 2001). Similarly, changes in this prevalence can be explained by how much the magnitude of this association changed over this period. For example, whether education became a stronger predictor of past-year dental visits in 2012 than in 2001. Decomposition analysis allows one to quantify the extent to which educational attainment (and other population characteristics) contributes to the observed changes in the prevalence of past-year dental visits. Improving trends within Mexico’s educational landscape warrant better understanding of how educational attainment impacts the ability of Mexican older adults to receive dental care.

While evidence suggests that education and insurance can lead to greater oral health utilization among older adults, few studies have explored how access to those resources and the prevalence of dental visits have shifted over time. This is particularly relevant in the case of Mexico as the Mexican government provided universal dental care benefits through *Seguro Popular*. One decade following the implementation of *Seguro Popular*, older adults in 2012 were more likely to have accessed any form of healthcare while also having acquired more education than a baseline wave of older adults in 2001. Thus, we might expect to find that changes in oral health access are a result of these changes in the population composition. In addition, oral health access might have also improved due to stronger statistical associations between education and dental visits, and insurance and dental visits over that same period. Together, these conditions enhance access to oral health services for incoming cohorts of older adults. Given that education and access to health insurance are the main factors associated with oral health, we hypothesize that past-year dental visits are positively associated with (1) higher educational attainment and (2) health insurance. We also speculate that changes in the population composition and covariate effects contributed to an increased prevalence of dental care access between 2001 and 2012.

## Methods

### Data

The Mexican Health and Aging Study (MHAS) is a prospective panel study of adults aged 50 years and older from rural and urban areas across all 32 states in Mexico. MHAS collected data on sociodemographic characteristics, health conditions, aging, migration, and family networks. All questionnaires were administered in-person by trained interviewers from the Instituto Nacional de Estadística y Geografía (INEGI) of Mexico. The first three waves of the study were conducted in 2001, 2003, and 2012 (Wong et al., 2017). The 2001 baseline survey gathered information from a nationally representative sample of Mexican adults who were born before 1952. Households with at least one resident 50 years or older were randomly selected to participate in the study. Six states accounting for 40% of all migrants to the United States were over-sampled. The 2001 sample size consisted of direct interviews (*n* = 14,154) and proxy interviews with other household members (*n* = 1032) with a response rate of 91.8%. Follow-up interviews for this same cohort were conducted in 2003, 2012, 2015, and 2018. A 93.3% response rate resulted in a sample size of 14,250 individuals who participated in the 2003 survey. Last, the 2012 MHAS wave consisted of eligible participants from the 2003 follow-up sample (*n* = 14,283), new participant spouses (*n* = 385), and an added cohort of 6259 participants within the 50–59 age range (those born between 1952 and 1962). The response rate for the third wave was 88.1% with a total sample size of 18,465 respondents. This study focused on data from baseline and 2012.

### Exclusion Criteria

Analytic data were restricted to direct interviews with participants aged 50 and older at the time of data collection and focused on outcomes from the 2001 and 2012 samples (*n* = 12,432 for the 2001 wave and *n* = 13,636 for the 2012 wave). In the 2001 analytic sample, 55 observations (0.4%) were excluded due to missing data on the outcome variable and key predictors, resulting in a sample size of 12,377 participants. The same exclusion criteria were applied to the 2012 sample. Out of 13,636 eligible respondents in 2012, 200 observations (1.5%) were dropped for having incomplete responses on key predictors and the dependent variable. The final analytic sample size for the 2012 analysis was 13,436 participants.

### Measures

Having at least one past-year oral health visit was the outcome variable in this analysis. This variable was captured from both 2001 and 2012 questionnaires with the item, “In the last year, about how many times have you seen a dentist?” This variable was consolidated into a binary variable (0 indicates no dental visits and 1 represents at least one oral visit) since only about 6% of each sample had exactly two dental visits in a year, fewer than 5% had three dental visits in a year, and fewer than 5% of participants in each cohort had four or more past-year dental visits.

The following predictors were used in the 2001 and 2012 analyses: age, gender, years of education, health insurance status, net worth, urban residence, current smoking status, number of chronic conditions, mobility limitations, and having five or more depressive symptoms (Castrejón-Pérez et al., 2012; Ramírez et al., 2011; Sánchez-García et al., 2007). Age in years was a continuous variable that was gathered from the demographic section of the survey. Gender was captured as a dichotomous categorical variable from the demographic section of both 2001 and 2012 questionnaires. Response options were “Male” or “Female.” Educational attainment was operationalized as the number of years of completed education by respondents in both survey waves. For this analysis, education was recoded from a continuous variable into a 5-item categorical variable with the following groupings: “0 years of education,” “1–5 years,” “6 years,” “7–9 years,” and “10 or more years.” Next, health insurance included the following options of insurance programs in Mexico: Social Security/Instituto Mexicano del Seguro Social, Institute for Social Security and Services for State Workers/ISSSTE, Pemex/Defensa/Marina, private insurance, or other types of insurance. Respondents without any health insurance were coded as “0” and respondents with any form of public or private insurance were coded as “1.”

We used an indicator of wealth constructed by the MHAS team (see Wong et al., 2007). Net worth is based on the monetary value of all assets (including businesses, land, housing, stocks and bonds, and savings) minus debts for individuals (or for the couple if the respondent was married/cohabiting). The MHAS project estimated wealth values while imputing missing values in the components of wealth with the method of sequence regressions (see Wong et al., 2007). The imputation method has several advantages: allowing variable values to be zero, accounting for other imputed variables, and incorporating responses based on categorical responses (“unfolding brackets”). We use four categories of wealth based on quartiles of the distribution. For stratified analysis, the wealth categories were created by gender and age. Next, urban place of residence was a dichotomous variable that defined urbanicity as a primary locality with a population of 15,000 people or more (Salinas et al., 2010). Lifestyle risk factors such as current smoking were defined as “currently smokes cigarettes” (Wong et al., 2008).

The next domain of predictors represents health variables that have an inverse association with past-year dental visits. The chronic conditions variable was a composite of seven self-reported items that indicated “yes” to having been diagnosed with any of the following conditions: Hypertension, diabetes, cancer, respiratory problems, heart problems, stroke, and arthritis. The number of chronic conditions was summed for each respondent, then recoded into a 3-item categorical variable (Díaz-Venegas et al., 2017). Response categories were “None” for individuals with no diagnosis of chronic disease, “One” for listing one chronic disease, and “Two or more” for individuals with at least two chronic conditions.

Next, the mobility limitations variable was collected as a series of nine self-reported items related to activities of daily living. The mobility limitation index was constructed according to Long and Pavalko (2004). The questionnaire item asked, “Please tell me if you have any difficulty in doing each of the daily activities that I am going to read. Do not include difficulties that you believe will last less than 3 months.” Response options were “yes,” “no,” “can’t do,” doesn’t do,” “refused,” or “don’t know” for the following activities: Walking several blocks, walking one block, sitting for 2 hours, climbing several flights of stairs without resting, stooping/kneeling/crouching, extending arms above shoulder level, pushing or pulling large objects like a chair, lifting or carrying objects that weigh over 5 kg, and picking up a 1-peso coin from a table. In our analysis, the mobility variable was recoded as a binary variable (yes or no). Individuals that answered “no” to any of these items were coded as having no mobility limitations, and those who responded “yes,” “can’t do,” or “doesn’t do” were coded as having one or more mobility limitations.

Last, depressive symptoms were based on the mental health index used by Torres and Wong (2013). The number of depressive symptoms was a composite score calculated from nine questions with a “yes” or “no” response. Each item asked whether a majority of the time respondents felt the following during the past week: Depressed, everything was an effort, restless sleep, happy, lonely, enjoyed life, sad, tired, and had a lot of energy. Items with a “yes” response for “depressed,” “effort,” “restless sleep,” “lonely,” “sad,” and “tired” were coded as “1.” “Happy,” “enjoyed life,” and “had a lot of energy” were reversed coded so that “no” responses were coded as “1.” All nine items were summed into a composite score with a range of 0–9. The depression score was then converted into a binary variable by using a value of one for those with a score of five or more or zero otherwise (Torres & Wong, 2013).

### Statistical Analyses

Univariate analyses were conducted for each wave to assess the distributions of each predictor (age, education, net worth, health insurance, urban residence, smoking status, chronic conditions, mobility limitations, and depressive symptoms) and the outcome variable (having at least one past-year dental visit) (Table 1). As shown in Table 1, the population composition by education significantly differs by age and sex between 2001 and 2012. For example, there is a higher fraction of people with 7+ years of education in 2012 than in 2001 among people aged 50–54 for both women and men, but a non-significant difference in 7+ years of education over time among women aged 65+. Similarly, there is a statistically significantly higher fraction of insured (and lower fraction of non-insured) population in 2012. We thus conducted stratified analysis by gender (female and male) and age group (50–64 years and 65 years and above) to assess the differential impact of educational attainment in these population subgroups. Binary logistic regressions evaluated the association between past-year dental visits and the selected predictors in each wave. Variables were incrementally added to each statistical model. Goodness-of-fit was assessed across the logistic regression models using log likelihood-ratio tests, Akaike’s Information Criteria (AIC), and Bayesian Information Criteria (BIC). The final logistic regression analyses included all predictors (referred to as the full model).

**Table 1.** Baseline characteristics from 2001 (*n* = 12,377) and 2012 (*n* = 13,436) MHAS samples*.

	Aged 50–64				Aged 65+			
	2001	2012	Change 2001–2012		2001	2012	Change 2001–2012	
Variable	% [1]	% [2]	[2−1]	*p*-value	% [1]	% [2]	[2−1]	*p*-value
**Female**								
One or more dental visits in the past year (dependent variable)	31.0	39.8	8.8	<.001	21.2	29.5	8.3	<.001
Age** mean years (SD)	56.3 (4.3)	57.4 (4.2)	1.1	<.001	72.7 (6.4)	73.2 (6.6)	0.5	.015
Years of education								
0 years	18.1	10.9	−7.2	<.001	32.4	25.0	−7.4	<.001
1–5 years	36.0	26.6	−9.4	<.001	37.8	38.1	0.3	.854
6 years	20.6	24.7	4.1	<.001	14.8	18.1	3.3	.003
7–9 years	17.3	23.9	6.6	<.001	10.9	12.7	1.8	.063
10 or more years	8.0	14.0	6.0	<.001	4.1	6.1	2.0	.003
Insurance type								
No insurance	32.2	10.6	−21.6	<.001	30.5	8.9	−21.6	<.001
Private or other insurance	4.8	4.6	−0.2	.671	3.6	3.7	0.1	.070
Seguro popular (in 2012 only)		29.8				25.6		
ISSSTE/PEMEX employers	17.4	17.8	0.4	.611	22.5	21.2	−1.3	.289
IMSS/social security	51.3	46.9	−4.4	<.001	51.4	52.6	1.2	.430
Net worth								
1st quartile	22.2	23.5	1.3	.153	32.2	26.9	−5.3	<.001
2nd quartile	25.8	26.4	0.6	.512	22.2	23.0	0.8	.486
3rd quartile	26.2	25.7	−0.5	.663	23.9	25.1	1.2	.354
4th quartile	25.9	24.3	−1.6	.111	21.7	24.9	3.2	.012
Urban residence	71.1	72.6	1.5	.152	71.1	73.8	2.7	.039
Current smoker	10.8	9.1	−1.7	.012	7.3	4.4	−2.9	<.001
Number of chronic conditions								
None	34.9	36.6	1.7	.109	26.6	23.6	−3.0	.017
One	38.1	36.1	−2.0	.071	37.7	38.6	0.9	.508
Two or more	27.0	27.3	0.3	.805	35.7	37.8	2.1	.151
Has 5 or more depressive symptoms	40.2	37.9	−2.3	.031	47.6	42.1	−5.5	<.001
Has at least one mobility limitation	65.0	66.8	1.8	.098	79.8	83.5	3.7	.001
**Male**								
One or more dental visits in the past year (dependent variable)	26.7	32.3	5.6	<.001	19.7	28.0	8.3	<.001
Age** mean years (SD)	56.2 (4.2)	57.9 (4.3)	1.7	<.001	72.7 (6.4)	73.1 (6.7)	0.4	.054
Years of education								
0 years	14.1	7.5	−6.6	<.001	29.4	20.4	−9.0	<.001
1–5 years	30.8	21.7	−9.1	<.001	40.7	35.9	−4.8	.001
6 years	22.7	22.3	−0.4	.727	14.3	20.6	6.3	<.001
7–9 years	14.2	21.0	6.8	<.001	6.9	10.3	3.4	<.001
10 or more years	18.2	27.5	9.3	<.001	8.8	12.9	4.1	<.001
Insurance type								
No insurance	38.1	15.6	−22.5	<.001	35.1	10.9	−24.2	<.001
Private or other insurance	5.0	5.4	0.4	.569	4.0	4.0	0.0	.942
Seguro popular (in 2012 only)		28.0				25.6		
ISSSTE/PEMEX employers	15.0	15.8	0.8	.425	16.8	18.7	1.9	.098
IMSS/social security	46.0	44.7	−1.3	.310	50.9	51.4	0.5	.767
Net worth								
1st quartile	19.7	23.7	4.0	<.001	23.7	21.5	−2.2	.083
2nd quartile	25.0	24.9	−0.1	.931	25.7	25.2	−0.5	.729
3rd quartile	26.0	26.0	0.0	.971	25.3	25.3	0.0	.949
4th quartile	29.4	25.4	−4.0	.001	25.3	28.1	2.8	.048
Urban residence	69.5	70.8	1.3	.270	63.1	67.8	4.7	.001
Current smoker	29.5	22.6	−6.9	<.001	20.6	14.2	−6.4	<.001
Number of chronic conditions								
None	55.6	53.2	−2.4	.072	41.2	39.2	−2.0	.176
One	29.8	30.4	0.6	.648	34.9	34.8	−0.1	.920
Two or more	14.6	16.4	1.8	.059	23.9	26.0	2.1	.107
Has 5 or more depressive symptoms	20.2	17.9	−2.3	.020	30.2	24.6	−5.6	<.001
Has at least one mobility limitation	42.6	42.1	−0.5	.674	67.2	65.2	−2.0	.160

**p-values are estimated from logistic regressions, except for age for which OLS is used, taking into account the complex survey design (sampling weights).

Source: Mexican Health and Aging Study (MHAS), 2001–2012.

*Percentages are weighted to reflect the national Mexican older adult population in 2001 and 2012, respectively: weighted *N* in 2001= 778,055; *N* in 2012= 905,069.

### Decomposition Analysis (Using the Oaxaca–Blinder Procedure)

The Oaxaca–Blinder procedure is a regression-based decomposition analysis that allow us to explore differences in the prevalence of past-year dental visits between the 2001 and 2012 MHAS. This procedure assessed how changes in the prevalence of past-year dental visits between 2001 and 2012 were explained by (1) changes in the composition or *endowment* of the predictors within each cohort and (2) changes in the *impact* of each coefficient from each cohort (Jann, 2008). The following equation displays how the population endowment and impact of each covariate contributed to the difference in the prevalence of past-year dental visits between cohort years:

(1) $$D=\;\Delta Age\beta_{At_{0}}+\;\Delta Education\beta_{Et_{0}}+\;\Delta Insurance\beta_{It_{0}}+\;\Delta Health_{Ht_{0}}+\;Age_{t_{0}}\Delta \beta_{A}+\;Education_{t_{0}}\Delta \beta_{E}+\;\;Insurance_{t_{0}}\Delta \beta_{I}+\;\;Health_{t_{0}}\Delta \beta_{H}+Constant+Unexplained$$

In equation (1), *D* represents the difference in the prevalence of past-year dental visits from 2001 to 2012, *t*_*0*_ represents the 2001 baseline wave, and Δ is the change between 2001 and 2012. The decomposition leads to additive coefficients for each variable. To simplify the presentation of results, we collapse) health variables into a single “health” domain (i.e., adding up coefficients from equation (1)). Full results from equation (1) are shown in Appendix 3. The decomposition shown in equation (1) partitions changes in the prevalence of past-year dental visits between 2001 and 2012 into two counterfactual scenarios. First, it holds the beta coefficients constant at their 2001 level (i.e., beta coefficients from the regression in 2001) while the population composition changes over time (first line of equation (1)), and second, it holds the population composition constant at its 2001 level while the beta coefficients change over time (second line of equation (1)). As it is always the case in regression approaches, there is a residual term which in this case corresponds to differences in the prevalence of past-year dental visits that are not explained by the predictors (third line of equation (1)). All analyses used sampling weights and were conducted using STATA version SE/15.1 statistical software.

There are different ways to interpret decompositions results, here we offer a general description and further elaborate it in the results section. In counterfactual analysis, we are interested in answering the hypothetical question “what if X had not changed, how much of the observed change in the prevalence of past-year dental visits is due to changes in Z.” Thus, in the case of educational attainment, one can interpret results from line 1 in equation (1) as follows: how much of the increase in the prevalence in past-year dental visits is due to changes in the fraction of people with an educational attainment between 2001 and 2012 if the impact of education on said prevalence remains constant over the time period. Similarly, line 2 from equation (1) would indicate: how much of the increase in the prevalence in past-year dental visits is due to changes in the impact of education between 2001 and 2012 if there had not been an increasing fraction of people with more education over time (i.e., the fraction of people by education remains constant at 2001-level over the time period).

## Results

### Descriptive Statistics

Table 1 displays baseline characteristics for the 2001 (*N* = 12,377) and 2012 (*N* = 13,436) MHAS data. From these results, we observed that past-year dental visits significantly increased between 2001 and 2012 across all gender and age groups. The prevalence of past-year dental visits was lowest among males aged 65 and older (19.7% in 2001 and 28.0% in 2012) and largest among females aged 50 to 64 (31.0% in 2001 and 39.8% in 2012). Males generally had a lower prevalence of past-year dental visits compared to females, but with a declining gap among the 65+ age group.

Regarding educational attainment, more participants attained higher levels of education in 2012 compared to 2001, particularly among younger older adults (aged 50–64). The largest increase was observed among males aged 50 to 64: the proportion of individuals in this group who attained 10 or more years of education increased from 18.2% in 2001 to 27.5% in 2012. Higher levels of education were consistently lowest among females versus males and individuals aged 65 and older compared to the 50 to 64 age group.

Trends in insurance status showed that a significantly larger proportion of the population was insured in 2012 compared to 2001, with a larger increase among people aged 65+. Across this period, insurance rates among 50- to 64-year-old females rose from 67.8% to 89.4%, and among 65+ females insurance status increased from 69.5% to 91.1%. Among males aged 50- to 64- and 65+, insurance status between cohorts increased from 62.0% to 84.4% and 64.9% to 89.2%, respectively. Importantly, the distribution of net worth in 2012 moved to the left among people aged 50–64 and to the right among those aged 65+ relative to 2001. This implied that there was a smaller fraction of people at the higher quartiles of net worth in 2012 among younger adults (fourth quartile: 25.9% in 2001 vs. 24.3% in 2012), but the opposite is true among older adults (fourth quartile: 21.7% in 2001 vs. 24.9% in 2012). Similarly, the prevalence of health conditions significantly increased over time. Depressive symptoms, having two or more chronic conditions, and having mobility limitations all had the highest prevalence among the 65+ age groups versus the lower age category and females compared to males.

### Multiple Logistic Regression

Odds ratios from binary multiple logistic regressions by age group, period and sex are displayed in Table 2. These findings show that education has the strongest association with increased odds of having a past-year dental visit across all subgroups. These trends occurred along a gradient, illustrating that the likelihood of having a past-year dental visit increased incrementally between each educational category from 0 to 10+ years of education. Similar results were found with higher levels of net worth. We also found significant and marginally significant (*p*-values below .06) associations between having insurance and receiving a past-year dental visit though results were less significant in 2012. Receiving past-year dental care was also more likely among non-smokers versus current smokers (except among 50–64 year-old females in 2001, in which the opposite result was observed) and more prevalent among those who live in urban versus rural areas.

**Table 2.** Weighted logistic regression results: Odds ratios of having a past-year dental visit among Mexican adults aged 50 and older by age group and survey year.

	Aged 50–64		Aged 65+	
Variable	2001	2012	2001	2012
**Female**				
Age	0.99	.98*	.96*	.97**
Years of education				
0 years (reference)				
1–5 years	1.23+	1.35+	1.28	1.06
6 years	1.63**	1.47*	1.83*	1.56*
7–9 years	2.61**	1.95**	2.75**	2.26**
10 or more years	3.43**	3.92**	2.90**	3.67**
Net worth				
1st quartile (reference)				
2nd quartile	1.27+	1.13	1.01	1.15
3rd quartile	1.30+	1.11	1.02	1.19
4th quartile	1.71**	1.36*	1.29	1.25
Insurance status				
No insurance (reference)				
Has insurance	1.24+	1.46*	1.69*	1.34
Urban residence				
No (reference)				
Yes	1.50**	1.53**	1.34	1.30+
Current smoker				
No (reference)				
Yes	1.28+	1.06	0.77	0.65+
Number of chronic conditions				
None (reference)				
One	1.08	1.03	1.09	1.00
Two or more	1.29+	1.03	1.19	1.23
Has at least one mobility limitation				
No (reference)				
Yes	.96	1.13	1.10	0.83
Has 5 or more depressive symptoms				
No (reference)				
Yes	1.17+	.90	1.16	.98
Constant	1.06	2.62+	3.67	6.01*
Analytic sample size	4406	4298	2299	3396
Population sample size	271,174	287,810	119,123	202,724
**Male**				
Age	.99	1.01	.98+	.98*
Years of education				
0 years (reference)				
1–5 years	1.87**	1.47	1.58*	1.64**
6 years	1.91**	1.73+	2.13**	1.80**
7–9 years	2.64**	2.44**	2.57**	2.61**
10 or more years	3.95**	3.29**	3.05**	3.51**
Net worth				
1st quartile (reference)				
2nd quartile	1.15	1.18	1.00	1.03
3rd quartile	1.25	1.14	1.05	1.11
4th quartile	1.89**	1.80**	1.65*	1.38+
Insurance status				
No insurance (reference)				
Has insurance	1.31*	1.26	1.38+	1.30
Urban residence				
No (reference)				
Yes	1.24+	1.50**	1.32	1.32+
Current smoker				
No (reference)				
Yes	.96	.95	.70+	.73+
Number of chronic conditions				
None (reference)				
One	1.33*	1.41**	0.99	1.39*
Two or more	1.58**	1.35+	1.32	1.39*
Has at least one mobility limitation				
No (reference)				
Yes	.99	1.08	1.34+	1.11
Has 5 or more depressive symptoms				
No (reference)				
Yes	1.25+	.80	1.40+	.91
Constant	.82	.42	1.46	2.53
Analytic sample size	3587	2852	2085	2890
Population sample size	254,913	214,122	132,845	200,413

Source: Mexican Health and Aging Study (MHAS), 2001–2012. ** *p* < .001, * *p* < .01, + *p* < .05.

Results for the number of chronic conditions, mobility limitations and depressive symptoms show no statistically significant associations with past-year dental visits, except for a few cases. For example, having more chronic conditions increases the odds of past-year dental visit among males but not among females. Additionally, in contrast to the 2012 wave, groups from 2001 had greater odds (between 17% and 40%) of visiting a dentist if they had five or more depressive symptoms.

### Decomposition Results: Differences in Prevalence of Past-Year Dental Visits Between 2001 and 2012

The Oaxaca–Blinder decomposition results by sex and age group are shown in Table 3 (results for each individual covariate are shown in the Appendix1,2,3). Panel (A) in the table shows the prevalence of past-year dental visits in each year and the total change over time, which is shown to have increased over time. Panels (B) and (C) show how much of the increase in this prevalence is attributed to changes in the composition of the covariates while holding the impact of the covariates constant over time, changes in the effects of the covariates while holding the population composition constant at 2001-levels, respectively. The sum of the individual components in panels (B) and (C) equals the total change in the prevalence of oral health visits shown in panel (A).

**Table 3.** Weighted Oaxaca–Blinder decomposition results: Assessing the composition and effect of each predictor in the prevalence of past-year dental visits between the 2001 and 2012 in MHAS waves.

	Aged 50–64	Aged 65+
**Female**			
A) prevalence	% With a past-year dental visit	% With a past-year dental visit
2001	31.02	21.17
2012	39.78	29.52
Total change [2012–2001]	8.77	8.35
B) Effects of changes in the composition of covariates in 2012 versus 2001 (%)			
Age	−0.51	−0.36
Education	2.43	1.07
Net worth	−0.10	0.20
Insurance	1.77	1.20
Current smoking	−0.02	0.24
Urban	0.13	0.13
Health domain	0.09	−0.03
C) Effects of the changes in the effect of the covariates in 2012 versus 2001 (%)			
Age	−13.46	11.37
Education	−1.11	−2.01
Net worth	−2.24	0.97
Insurance	3.13	−3.79
Current smoking	−0.37	−0.14
Urban	0.41	−0.40
Health domain^1^	−1.40	−5.70
Constant	20.49	5.50
Unexplained	−0.47	0.10
**Male**			
A) prevalence	% With a past-year dental visit	% With a past-year dental visit
2001	26.74	19.68
2012	32.33	27.97
Total change (2012–2001)	5.59	8.28
B) Effects of changes in the composition of covariates in 2012 versus 2001 (%)			
Age	0.16	−0.20
Education	2.54	1.70
Net worth	−0.44	0.15
Insurance	0.99	1.10
Current smoking	0.07	0.36
Urban	0.10	0.22
Health domain^1^	0.23	0.17
C) Effects of the changes in the effect of the covariates in 2012 versus 2001 (%)			
Age	14.82	−1.73
Education	−2.97	−0.02
Net worth	−0.52	−0.24
Insurance	−0.67	−0.94
Current smoking	−0.07	0.11
Urban	2.86	−0.03
Health domain	−1.20	−1.67
Constant	−9.58	9.88
Unexplained	−0.73	−0.58

Source: Mexican Health and Aging Study (MHAS), 2001–2012.

^a^ Health domain includes adding decomposition coefficients from equation (1) for the number of chronic conditions, mobility limitations, and depressive symptoms.

#### Population Composition

For both females and males, changes in the fraction of the population with education and insurance had the largest positive impact in explaining the increasing prevalence of a past-year dental visit over time. The magnitude of this impact is generally larger among younger cohorts (aged 50–64). To simplify the description of results, we focus first among women aged 50–64 and then generalize the findings. In this age group, there was a higher fraction of people with more education in 2012 which accounted for about 2.43 percentage points of the 8.77% increase in the prevalence of having had a past-year dental visit between 2001 and 2012. Similarly, a higher fraction of people with health insurance in 2012 among women aged 50–64 accounted for an additional 1.77 percentage points of the 8.77% change in the prevalence past-year dental visits. Thus, the fact that health insurance became more available in recent years led to an increase in the fraction of people who had a past-year dental visit in recent times. Interestingly, results also indicate there was a negligible change in the prevalence of health conditions and in the fraction of people living in urban areas among women aged 50–64 between 2001 and 2012; thus, these covariates accounted for a very small magnitude of the increase in the prevalence of a past-year dental visits, 0.13 and 0.09 percentage points, respectively. In contrast, changes in the age structure of the population and in the net worth distribution contributed to a *reduction* (negative sign) in the prevalence. This suggests that had the population not become older, or with a lower population fraction in the upper quartiles of the net worth distribution, the observed increase in the prevalence of a past-year dental visit would have been slightly higher by about 0.51 and 0.10 percentage points, respectively, than the observed change of 8.77 % (assuming the effect of the other covariates remained stable between 2001 and 2012).

Results for women aged 65+ are similar to those aged 50–64. For example, had the population not become older in 2012 than 2001, the increase of 8.35% in the prevalence would have been higher by about 0.36 percentage points. Nonetheless, due to an increasing fraction of older adult women with higher education, with access to health insurance, or in the upper quartiles of the net worth distribution, the prevalence went up by about 1.07, 0.20, and 1.2 percentage points, respectively. Results for men are similar to those among women. For example, changes in educational attainment had the largest contribution to the increasing prevalence of past-year dental visits for both younger and older adult males, followed by positive contributions from all other covariates, and negative contribution from net worth among those aged 50–64 but positive contribution to those aged 65+ due to the changing net worth distribution over time.

#### Effect of Covariates

Although compositional changes had some relevance in explaining changes in the prevalence of a past-year dental visit, the largest magnitudes in the contributions came from increases in the effect of the covariates. These results can be interpreted as how much of the increase in the prevalence in past-year dental visits is due to changes in the impact of the covariates between 2001 and 2012 if the population composition remained as it was in 2001. That is, a negative sign in the contribution of effect of covariates indicates that the association of a covariate with past-year dental visits is less strong in 2012 than in 2001; in contrast, a positive sign in the contribution suggests a stronger association in 2012 than in 2001. In other words, a given covariate is more (less) predictive of past-year dental visits in 2012 if its contribution is positive (negative). For instance, the association of age with past-year dental visits varies between women and men and between younger and older adults. This association is less strong in 2012 than in 2001 among women aged 50–64 (negative sign), but the opposite is true among those aged 65+. In contrast, among men, age has a stronger association with past-year dental visits in 2012 in younger adults and a lesser one among those aged 65+. These results indicate that a stronger age association with past-year dental visits among older women and younger adult men contributed to about 11.37 and 14.82% points to their corresponding prevalence increase. In contrast, had the association with age being stronger in 2012 among women aged 50–64 and men aged 65+, the prevalence of past-year dental visits would have increased by about 13.46 and 1.73% points, respectively.

The impact of education, net worth, insurance, and health conditions is consistently less strong in 2012 among men, but among women these impacts vary by age group. For example, for women aged 65+, net worth is more predictive of past-year dental visits in 2012 contributing to an increase in the prevalence of about 0.97% points. Nonetheless, all other covariates show a less strong association in 2012 suggesting that had their link with past-year dental visits be more predictive in 2012, we would have observed an increase of about 11.58% points in past-year dental visits from education (2.01), insurance (3.79), and health conditions (5.79), respectively (11.58 = 2.01+ 3.79 + 5.79). Interestingly, access to insurance led to a stronger association with past-year dental visits in 2012 only among women aged 50–64 (with a 3.13% point contribution). For the other age groups, insurance had a lower impact on the prevalence in 2012 than in 2001.

#### Summary

Ultimately, a major demographic shift occurred between 2001 and 2012 in which Mexican older adults from 2012 attained higher levels of education and had higher insurance enrollment than the baseline wave. This change in population structure contributed to an increase in the prevalence of dental visits from 2001 to 2012. Nonetheless, population aging and the net worth distribution dragged down some of the progress and if it had not been for these population changes, the prevalence of past-year dental visits could have been higher. The role of the covariate effects by education and insurance were less consistent. In most age and gender groups, the covariate effects of insurance and education became less strong in 2012, except for women aged 50–64 for which insurance was a stronger predictor of past-year dental visits in 2012. Age also play a major role with stronger impact on the prevalence among women aged 65+ and among men aged 50–64.

## Discussion

In this paper, we study the association between socioeconomic and health factors on the prevalence of past-year dental visits among older Mexican adults in 2001 and 2012. As a result of major changes in health policies in the country during the period of study and the continuing aging of the Mexican population, we hypothesize that past-year dental visits are positively associated with higher educational attainment and health insurance. We report three main findings. First, in both 2001 and 2012, there were significant associations between higher educational attainment and past-year dental visits. Second, insurance status was also positively associated with past-year dental visits in both years. Third, the prevalence of past-year dental visits increased between 2001 and 2012 and was partly explained by changes in the population composition and also be changes in the association with the covariates, most notably by the covariate effect of age. For example, changes in the population composition, in which more individuals had access to insurance and higher education in 2012 compared to 2001, contributed to the shift in the prevalence of past-year dental visits.

Our results illustrate a significant positive association between educational attainment and past-year dental visits. This is consistent with previous literature, for example, results from 2001 and 2012 show that older Mexican adults with higher educational attainment were more likely to use any type of dental service in the past year compared to those with fewer years of education (Almeida et al., 2017; Andrade et al., 2020). Our findings further elucidate this link by showing that the increasing prevalence of having a past-year dental visit is in part due to the underlying changes in the population composition of Mexican adults who are achieving older age having attained higher levels of education than previous generations. Moreover, our decomposition results further elucidate that the association between education and past-year dental visits attenuated over time so the impact of education was less important in explaining the increasing prevalence of dental visits. Thus, our findings may suggest that dental care utilization is promoted by the advantages in knowledge and resources that are made more accessible through higher levels of education.

Dental care utilization had differential effects by gender and age group. Compared to adults aged 50–64 years, adults aged 65+ years had an overall lower proportion of dental visits. Males above age 64 had the lowest proportion of dental visits. Similar to findings from a longitudinal study of older adults in Germany (Spinler et al., 2019), our results revealed that female participants were consistently more likely than males to have a past-year dental visit, but the prevalence of dental visits among females decreased at a faster rate than males with older age. These data suggest that although females may have previously had more dental care access or better oral health-seeking behaviors compared to males, other unmeasured factors such as changes in employment status, widowhood, or low social support may lead to increasing disparities in dental care access among females later in life.

From our decomposition results, it was noted that the prevalence of past-year dental visits across all age and gender subgroups increased between 2001 and 2012. We found that changes in the population composition, specifically greater proportions of educational attainment and those with insurance, within this period partly explained this increase in the prevalence of dental visits. As expected, a larger proportion of older adults had insurance following the enactment of *Seguro Popular* in 2002. These findings suggest that the expansion of resources such as education and public health programs became more accessible in later generations and ultimately, contributed to improvements in dental care access and utilization. However, the covariate effects of education and insurance did not increase between 2001 and 2012. These effects declined because of the dominating contributions of the impact of age, which was found to be associated with a reduction in the prevalence of a dental visit. Nonetheless, the demographic shift towards higher education and insurance status still had an important role in increasing the prevalence of past-year dental visits over that decade.

This study adds to the limited body of knowledge on the impact that insurance and education have on oral health care access in aging populations. No other studies have assessed this topic using decomposition techniques to compare the impact of population composition from the associations with known risk factors between different cohorts of older adults. In particular, the enactment of *Seguro Popular* between each cohort wave was a notable event in Mexico’s history that transformed access to health services in general, and dental services in particular, across the country. Despite the comprehensive provision of oral healthcare in Mexico, our results showed that still, fewer than 40% of the Mexican population over age 50 received any dental care in the past 12 months (Table 1). Disparities in oral healthcare by age and gender also persist. Tailoring public health programs to bridge these gaps could lead to immediate and long-term benefits that effectively, improve rates of access to oral health care among older adults.

This study has some limitations. First, all measures relied on self-reported data. Recall bias and stigma may have influenced responses about sensitive health topics. To minimize bias, trained researchers followed a uniform methodology to gather in-person information at each respondent’s household. Second, the variable we constructed to assess chronic conditions captures only respondents who are aware of having a condition and may, therefore, undercount undiagnosed cases who have not received any recent care. Third, the operationalization of past-year dental visits entails any form of oral health treatment. Differentiating between preventive and emergency oral health treatment in future analyses would better illustrate the need for specific oral health services. Last, the MHAS questionnaire did not have any indicators for oral health morbidities such as tooth decay, gum disease, and tooth loss or whether a past-year dental visit was for a preventive or reactive care. We are thus unable to further analyze dental care more broadly. Although important, inclusion of oral health condition variables in addition to oral health utilization requires extending an already lengthy survey questionnaire.

Poor oral health is a global disease burden that could be mitigated by routine utilization of preventive or emergency dental visits. Disparities in access to dental care are readily seen among Mexican older adults with lower educational attainment and lack of health insurance. Especially in the midst of the COVID-19 pandemic, the disruption of dental services has burdened Mexican oral health providers and patients (Casillas Santana et al., 2021). Although new sterilization procedures prevent risk of COVID-19 infection, many dental providers have less time and fewer resources to cater to their patient populations than before the pandemic (Casillas Santana et al., 2021). Among the 20% of Mexicans that reside in rural communities, issues of dental care access were already exacerbated by provider shortages (The World Bank, 2018). In 2013, the provider–patient ratio for the general Mexican population was only 12 dentists per 100,000 population (Malmö University & World Health Organization, n.d.). Although this study did not specifically examine provider shortages as a form of access to dental care, future work can pursue ways to support the oral health workforce as they respond to both existing barriers to access and new pandemic-related challenges.

Suggestions for future research include an assessment of universal dental care programs in Mexico and other countries. The impact that disabilities and social support have on oral health-seeking behaviors later in life is another critical area of study. In addition, updated surveillance instruments could collect information not only on oral health utilization, but also on key oral health outcomes such as dental caries, permanent tooth loss, and oral hygiene. Moreover, as the proportion of older Mexican adults residing in the United States is increasing, further research can explore the impact of migration on oral health utilization within aging migrant populations (Gonzalez-Barrera & Lopez, 2013). Oral health requires proper maintenance throughout the life course. Providing universal access to dental treatment and removing barriers to care are necessary for meeting the oral health needs of underserved older adults.

## Appendix 1 Cross tabulations of insurance categories in 2001 and 2012.

**Table:**

	Insurance type in 2001 (*n* = 12,377)				
	No insurance	Private or other insurance	ISSSTE/PEMEX	IMSS/social security	
No insurance	4694	0	0	0	
Private or other insurance		535	56	132	
ISSSTE/PEMEX			2005	472	
IMSS/social security				5786	
Total	4694	535	2005	5786	

	Insurance type in 2012 (*n* = 13,436)				
	No insurance	Private or other insurance	ISSSTE/PEMEX	IMSS/social security	Seguro popular (in 2012 only)
No insurance	1644	0	0	0	0
Private or other insurance		564	83	177	30
ISSSTE/PEMEX			2351	575	122
IMSS/social security				6327	392
Seguro popular (in 2012 only)					3880
Total	1644	564	2351	6327	3880

Source: Mexican Health and Aging Study (MHAS), 2001–2012.

## Appendix B. Full weighted logistic regressions (with 95% confidence intervals and p-values): Odds ratios of having a past-year dental visit among Mexican adults aged 50 and older by age group and survey year.

**Table:**

Variables						
	2001			2012		
	OR	95% CI	*p*-value	OR	95% CI	*p*-value
**Female (aged 50–64)**						
Age	0.99	[0.98, 1.00]	.349	.98*	[0.96, 1.00]	.022
Years of education						
0 years (reference)						
1–5 years	1.23	[0.98, 1.55]	0.072	1.35+	[1.05, 1.72]	0.018
6 years	1.67**	[1.27, 2.09]	<0.001	1.47*	[1.14, 1.88]	0.003
7–9 years	2.61**	[2.01, 3.38]	<0.001	1.95**	[1.51, 2.52]	<0.001
10 or more years	3.43**	[2.51, 4.68]	<0.001	3.92**	[2.94, 5.22]	<0.001
Net worth						
1st quartile (reference)						
2nd quartile	1.27+	[1.02, 1.58]	.033	1.13	[0.94, 1.37]	.186
3rd quartile	1.30+	[1.05, 1.62]	.018	1.11	[0.02, 1.34]	.275
4th quartile	1.71**	[1.38, 2.12]	<.001	1.36*	[1.12, 1.66]	.002
Insurance status						
No insurance (reference)						
Has insurance	1.24+	[1.05, 1.46]	.013	1.46*	[1.18, 1.82]	.001
Urban residence						
No (reference)						
Yes	1.50**	[1.25, 1.80]	<.001	1.53**	[1.31, 1.80]	<.001
Current smoker						
No (reference)						
Yes	1.28+	[1.03, 1.60]	.029	1.06	[0.84, 1.33]	.639
Number of chronic conditions						
None (reference)						
One	1.08	[0.91, 1.28]	.396	1.03	[0.88, 1.20]	.716
Two or more	1.29+	[1.06, 1.57]	.012	1.03	[0.86, 1.24]	.719
Has at least one mobility limitation						
No (reference)						
Yes	0.96	[0.82, 1.13]	.653	1.13	[0.98, 1.31]	.097
Has 5 or more depressive symptoms						
No (reference)						
Yes	1.17+	[1.00, 1.36]	.046	.90	[0.78, 1.04]	.162
Constant	1.06	[0.40, 2.84]	.900	2.62+	[1.00, 6.86]	.050
Analytic sample size	4406			4298		
Population sample size	271,174			287,810		
**Female (aged 65+)**						
Age	0.96*	[0.94, 0.99]	.001	0.97**	[0.96, 0.99]	<.001
Years of education						
0 years (reference)						
1–5 years	1.28	[0.94, 1.74]	.003	1.06	[0.85, 1.32]	.632
6 years	1.83*	[1.27, 2.66]	.001	1.56*	[1.20, 2.02]	.001
7–9 years	2.75**	[1.83, 4.14]	<.001	2.26**	[1.70, 3.00]	<.001
10 or more years	2.90**	[1.66, 5.07]	<.001	3.67**	[2.54, 5.30]	<.001
Net worth						
1st quartile (reference)						
2nd quartile	1.01	[0.72, 1.42]	.939	1.15	[0.91, 1.46]	.231
3rd quartile	1.02	[0.73, 1.43]	.900	1.19	[0.95, 1.49]	.139
4th quartile	1.29	[0.94, 1.79]	.120	1.25	[0.99, 1.58]	.063
Insurance status						
No insurance (reference)						
Has insurance	1.69*	[1.25, 2.29]	.001	1.34	[0.98, 1.82]	.062
Urban residence						
No (reference)						
Yes	1.34	[0.99, 1.80]	.056	1.30+	[1.06, 1.58]	.011
Current smoker						
No (reference)						
Yes	0.77	[0.49, 1.22]	.268	0.65+	[0.43, 0.98]	.041
Number of chronic conditions						
None (reference)						
One	1.09	[0.80, 1.48]	.575	1.00	[0.81, 1.24]	.988
Two or more	1.19	[0.86, 1.64]	.287	1.23	[0.99, 1.54]	.061
Has at least one mobility limitation						
No (reference)						
Yes	1.10	[0.81, 1.49]	.547	0.83	[0.67, 1.04]	.105
Has 5 or more depressive symptoms						
No (reference)						
Yes	1.16	[0.90, 1.50]	.241	.98	[0.83, 1.17]	.856
Constant	3.67	[0.72, 18.65]	.117	6.01*	[2.08, 17.32]	.001
Analytic sample size	2299			3396		
Population sample size	119,123			202,724		
**Male (aged 50–64)**						
Age	0.99	[0.97,1.01]	.484	1.01	[0.98, 1.03]	.626
Years of education						
0 years (reference)						
1–5 years	1.87**	[1.36, 2.56]	<.001	1.47	[0.97, 2.22]	.072
6 years	1.91**	[1.37, 2.67]	<.001	1.73+	[1.14, 2.62]	.010
7–9 years	2.64**	[1.85, 3.76]	<.001	2.43**	[1.61, 3.70]	<.001
10 or more years	3.95**	[2.78, 5.60]	<.001	3.29**	[2.18, 4.98]	<.001
Net worth						
1st quartile (reference)						
2nd quartile	1.15	[0.88, 1.50]	.309	1.18	[0.92, 1.52]	.201
3rd quartile	1.25	[0.97, 1.63]	.089	1.14	[0.89, 1.47]	.298
4th quartile	1.89**	[2.78, 5.60]	<.001	1.80**	[1.40, 2.32]	<.001
Insurance status						
No insurance (reference)						
Has insurance	1.31*	[1.09, 1.58]	.004	1.26	[0.99, 1.60]	.059
Urban residence						
No (reference)						
Yes	1.24+	[1.01, 1.51]	.035	1.50**	[1.22, 1.85]	<.001
Current smoker						
No (reference)						
Yes	0.96	[0.80, 1.15]	.680	.95	[0.77, 1.16]	.609
Number of chronic conditions						
None (reference)						
One	1.33*	[1.11, 1.61]	.003	1.41**	[1.17, 1.71]	<.001
Two or more	1.58**	[1.24, 2.03]	<.001	1.35+	[1.05, 1.74]	.020
Has at least one mobility limitation						
No (reference)						
Yes	0.99	[0.84, 1.19]	.982	1.08	[0.90, 1.29]	.421
Has 5 or more depressive symptoms						
No (reference)						
Yes	1.25+	[1.01, 1.55]	.036	.80	[0.63, 1.01]	.060
Constant	0.82	[0.26, 2.55]	.733	.42	[0.13, 1.37]	.149
Analytic sample size	3587			2852		
Population sample size	254,913			214,122		
**Male (aged 65+)**						
Age	0.98+	[0.96, 1.00]	.043	.98*	[0.96, 0.99]	.002
Years of education						
0 years (reference)						
1–5 years	1.58*	[1.13, 2.20]	.007	1.64**	[1.24, 2.16]	<.001
6 years	2.13**	[1.41, 3.22]	<.001	1.80**	[1.32, 2.45]	<.001
7–9 years	2.57**	[1.56, 4.26]	<.001	2.61**	[1.83, 3.73]	<.001
10 or more years	3.05**	[1.92, 4.84]	<.001	3.51**	[2.48, 4.97]	<.001
Net worth						
1st quartile (reference)						
2nd quartile	1.00	[0.69, 1.45]	.997	1.03	[0.79, 1.36]	.806
3rd quartile	1.05	[0.72, 1.52]	.795	1.11	[0.85, 1.46]	.429
4th quartile	1.65*	[1.15, 2.36]	.006	1.38+	[1.06, 1.80]	.017
Insurance status						
No insurance (reference)						
Has insurance	1.38+	[1.03, 1.85]	.034	1.30	[0.95, 1.77]	.103
Urban residence						
No (reference)						
Yes	1.32	[0.99, 1.77]	.061	1.32+	[1.07, 1.63]	.010
Current smoker						
No (reference)				A		
Yes	0.70+	[0.51, 0.96]	.028	0.73+	[0.56, 0.95]	.021
Number of chronic conditions						
None (reference)						
One	0.99	[0.74, 1.32]	.947	1.39*	[1.13, 1.71]	.002
Two or more	1.32	[0.95, 1.84]	.095	1.39*	[1.10, 1.77]	.006
Has at least one mobility limitation						
No (reference)						
Yes	1.34+	[1.01, 1.79]	.043	1.11	[0.91, 1.36]	.298
Has 5 or more depressive symptoms						
No (reference)						
Yes	1.40+	[1.06, 1.84]	.017	0.91		.400
Constant	1.46	[0.30, 7.16]	.643	2.53	[1.05, 1.72]	.098
Analytic sample size	2085			2890	[1.14, 1.88]	
Population sample size	132,845			200,413	[1.51, 2.52]	

Source: Mexican Health and Aging Study (MHAS), 2001–2012. ** *p* < .001, * *p* < .01, + *p* < .05.

## Appendix C. Full table of weighted Oaxaca-Blinder decomposition results: Assessing the composition and effect of each predictor in the prevalence of past-year dental visits between the 2001 and 2012 MHAS waves.

**Table:**

	Aged 50–64			Aged 65+		
**Female**						
Prevalence	Coefficient	Standard error	*p*-value	Coefficient	Standard error	*p*-value
2001	0.3102	0.0075	<.001	0.2117	.0097	<.001
2012	0.3978	0.0077	<.001	0.2952	.0083	<.001
Total change [2012–2001]	0.0877	0.0107	<.001	0.0835	.0127	<.001
Effects of changes in the composition of covariates in 2012 versus 2001						
Age	−0.0051	0.0022	0.023	−0.0036	0.0013	.006
Years of education						
0 years (reference)						
1–5 years	−0.0061	0.0026	.020	<0.0001	0.0002	.864
6 years	0.0034	0.0014	.013	0.0028	0.0013	.024
7–9 years	0.0095	0.0022	<.001	0.0028	0.0016	.075
10 or more years	0.0175	0.0028	<.001	0.0050	0.0018	.005
Sum of education coefficients	0.0243			0.0107		
Net worth						
1st quartile (reference)						
2nd quartile	0.0002	0.0003	.557	0.0002	0.0004	.546
3rd quartile	−0.0001	0.0002	.686	0.0004	0.0005	.431
4th quartile	−0.0010	0.0007	.156	0.0013	0.0009	.134
Sum of net worth coefficients	−0.0010			0.0020		
Insurance	0.0177	0.0049	<.001	0.0120	0.0059	.040
Smoking	−0.0002	0.0004	.645	0.0024	0.0013	.067
Urban	0.0013	0.0010	.166	0.0013	0.0008	.110
Health domain						
Has 5 or more depressive symptoms	0.0005	0.0005	.240	0.0002	0.0009	.856
Has at least one mobility limitation	0.0004	0.0004	.241	−0.0013	0.0009	.145
Has one chronic condition	−0.0001	0.0003	.721	<0.001	0.0002	.988
Has two or more chronic conditions	<0.0001	0.0001	.838	0.0008	0.0007	.256
Sum of health coefficients	0.0009			−0.0003		
Effects of the changes in the effect of the covariates in 2012 versus 2001						
Age	−0.1346	0.1456	0.358	0.1137	0.1633	0.481
Years of education						
0 years (reference)						
1–5 years	0.0049	0.0095	.603	−0.0129	0.0131	.324
6 years	−0.0054	0.0093	.560	−0.0052	0.0073	.476
7–9 years	−0.0145	0.0091	.110	−0.0045	0.0057	.425
10 or more years	0.0039	0.0064	.544	0.0026	0.0038	.499
Sum of education coefficients	−0.0111			−0.0201		
Net worth						
1st quartile (reference)						
2nd quartile	−0.0061	0.0081	.447	0.0053	0.0087	.538
3rd quartile	−0.0085	0.0078	.278	0.0068	0.0093	.464
4th quartile	−0.0116	0.0074	.119	−0.0016	0.0091	.863
Sum of net worth coefficients	−0.0224			0.0097		
Insurance	0.0315	0.0264	.232	−0.0379	0.1633	.290
Smoking	−0.0037	0.0031	.233	−0.0014	0.1756	.577
Urban	0.0041	0.0186	.834	−0.0040	0.0241	.867
Health domain						
Has 5 or more depressive symptoms	−0.0205	0.0085	.016	−0.0126	0.0118	.284
Has at least one mobility limitation	0.0225	0.0155	.145	−0.0410	0.0286	.152
Has one chronic condition	−0.0035	0.0090	.700	−0.0059	0.0130	.652
Has two or more chronic conditions	−0.0125	0.0077	.103	0.0025	0.0134	.854
Sum of health coefficients	−0.014			−0.0570		
Constant	0.2049	0.1481	.160	0.0550	0.1756	.766
Unexplained	−0.0047			0.0010		
**Male**						
Prevalence	Coefficient	Standard error	*p*−value	Coefficient	Standard error	*p*−value
2001	0.2674	0.0078	<.001	0.1968	0.0094	<.001
2012	0.3233	0.0090	<.001	0.2797	0.0087	<.001
Total change (2012−2001)	0.0559	0.0019	<.001	0.0828	0.0128	<.001
Effects of changes in the composition of covariates in 2012 versus 2001						
Age	0.0016	0.0033	.0627	−0.0020	0.001	.044
Years of education						
0 years (reference)						
1–5 years	−0.0066	0.0036	.069	−0.0041	0.0017	.015
6 years	−0.0004	0.0011	.729	0.0064	0.0021	.002
7–9 years	0.0115	0.0030	<.001	0.0058	0.0018	.001
10 or more years	0.0209	0.0043	<.001	0.0089	0.0025	<.001
Sum of education coefficients	0.0254			0.0170		
Net worth						
1st quartile (reference)						
2nd quartile	<0.001	0.0003	.931	<0.001	0.0001	.842
3rd quartile	<0.001	0.0003	.971	<0.001	0.0003	.949
4th quartile	−0.0044	0.0016	.006	0.0015	0.0010	.129
Sum of net worth coefficients	−0.0044			0.0015		
Insurance	0.0099	0.0050	.047	0.0110	0.0064	.084
Smoking	0.0007	0.0014	.609	0.0036	0.0017	.032
Urban	0.0010	0.0009	.289	0.0022	0.0011	.044
Health domain						
Has 5 or more depressive symptoms	0.0010	0.0007	.143	0.0009	0.0011	.410
Has at least one mobility limitation	−0.0001	0.0002	.709	−0.0004	0.0004	.402
Has one chronic condition	0.0004	0.0008	.651	−0.0001	0.0008	.921
Has two or more chronic conditions	0.0010	0.0007	.145	0.0012	0.0009	.164
Sum of health coefficients	0.0023			0.0017		
Effects of the changes in the effect of the covariates in 2012 versus 2001 (%)						
Age	0.1482	0.1767	.402	−0.0173	0.1645	.912
Years of education						
0 years (reference)						
1–5 years	−0.0109	0.0118	.355	0.0023	0.0137	.868
6 years	−0.0049	0.0125	.699	−0.0060	0.0092	.512
7–9 years	−0.0035	0.0120	.771	0.0003	0.0056	.964
10 or more years	−0.0104	0.0154	.500	0.0031	0.0067	.640
Sum of education coefficients	−0.0297			−0.0002		
Net worth						
1st quartile (reference)						
2nd quartile	0.0014	0.0097	.887	0.0015	0.0103	.882
3rd quartile	−0.0050	0.0010	.617	0.0026	0.0102	.802
4th quartile	−0.0025	0.0096	.797	−0.0086	0.0109	.429
Sum of net worth coefficients	−0.0052			−0.0024		
Insurance	−0.0067	0.0273	.808	−0.0094	0.0337	.786
Smoking	−0.0007	0.0065	.915	0.0011	0.0052	.840
Urban	0.0286	0.0029	.212	−0.0003	0.0215	.990
Health domain						
Has 5 or more depressive symptoms	−0.0169	0.0063	.008	−0.0182	0.0076	.017
Has at least one mobility limitation	0.0068	0.0114	.555	−0.0213	0.0197	.280
Has one chronic condition	0.0036	0.0088	.684	0.0204	0.0111	.067
Has two or more chronic conditions	−0.0055	0.0061	.370	0.0024	0.0093	.797
Sum of health coefficients	−0.0120			−0.0167		
Constant	−0.0958	0.1859	.610	0.0988	0.1739	.559
Unexplained	−0.0073			−0.0058		

Source: Mexican Health and Aging Study (MHAS), 2001−2012.

## References

[bibr1-08982643221086586] Almeida A. P. S. C. Nunes B. P. Duro S. M. S. Facchini L. A. (2017). Socioeconomic determinants of access to health services among older adults: A systematic review. Revista de Saúde Pública, 51, 50. 10.1590/s1518-8787.2017051006661 28513761 PMC5779074

[bibr2-08982643221086586] Andrade F. B. de Antunes J. L. F. Andrade F. C. D. Lima-Costa M. F. F. Macinko J. (2020). Education-related inequalities in dental services use among older adults in 23 countries. Journal of Dental Research, 99(12), 1341–1347. 10.1177/0022034520935854 32623932 PMC7580169

[bibr3-08982643221086586] Burr J. A. Lee H. J. (2013). Social relationships and dental care service utilization among older adults. Journal of Aging and Health, 25(2), 191–220. 10.1177/0898264312464497 23123482

[bibr4-08982643221086586] Casillas Santana M. Á. Martínez Zumarán A. Patiño Marín N. Castillo Silva B. E. Sámano Valencia C. Salas Orozco M. F. (2021). How dentists face the COVID-19 in Mexico: A nationwide cross-sectional study. International Journal of Environmental Research and Public Health, 18(4), 1750. 10.3390/ijerph18041750 33670181 PMC7916932

[bibr5-08982643221086586] Castrejón-Pérez R. C. Borges-Yáñez S. A. Gutiérrez-Robledo L. M. Ávila-Funes J. A. (2012). Oral health conditions and frailty in Mexican community-dwelling elderly: A cross sectional analysis. BMC Public Health, 12(1), 773. 10.1186/1471-2458-12-773 22971075 PMC3490998

[bibr6-08982643221086586] Castrejón-Pérez R. C. Jiménez-Corona A. Bernabé E. Villa-Romero A. R. Arrivé E. Dartigues J.-F. Gutiérrez-Robledo L. M. Borges-Yáñez S. A. (2017). Oral disease and 3-year incidence of frailty in Mexican older adults. The Journals of Gerontology: Series A, 72(7), 951–957. 10.1093/gerona/glw201 28329793

[bibr7-08982643221086586] Coll P. P. Lindsay A. Meng J. Gopalakrishna A. Raghavendra S. Bysani P. O’Brien D. (2020). The prevention of infections in older adults: Oral health. Journal of the American Geriatrics Society, 68(2), 411–416. 10.1111/jgs.16154 31479533

[bibr8-08982643221086586] Comisión Nacional de Protección Social en Salud . (2012). Catálogo universal de servicios de Salud 2012 (CAUSES). http://data.salud.cdmx.gob.mx/portal/seguro_popular/index/pdf/causes2012.pdf

[bibr9-08982643221086586] Díaz-Venegas C. Sáenz J. L. Wong R. (2017). Family size and old-age wellbeing: Effects of the fertility transition in Mexico. Ageing and Society, 37(3), 495–516. 10.1017/S0144686X15001221 28239210 PMC5321659

[bibr10-08982643221086586] Díaz-Venegas C. Samper-Ternent R. Michaels-Obregón A. Wong R. (2019). The effect of educational attainment on cognition of older adults: Results from the Mexican Health and Aging Study 2001 and 2012. Aging & Mental Health, 23(11), 1586–1594. 10.1080/13607863.2018.1501663 30449138 PMC6525654

[bibr11-08982643221086586] Furuta M. Yamashita Y. (2013). Oral health and swallowing problems. Current Physical Medicine and Rehabilitation Reports, 1(4), 216–222. 10.1007/s40141-013-0026-x 24392281 PMC3873078

[bibr12-08982643221086586] Gironda M. W. Maida C. Marcus M. Wang Y. Liu H. (2013). Social support and dental visits. The Journal of the American Dental Association, 144(2), 188–194. 10.14219/jada.archive.2013.0098 23372135 PMC9684585

[bibr13-08982643221086586] Gonzalez-Barrera A. Lopez M. H. (2013). A demographic portrait of Mexican-origin Hispanics in the United States. Pew Research Center. https://www.pewresearch.org/hispanic/2013/05/01/a-demographic-portrait-of-mexican-origin-hispanics-in-the-united-states/

[bibr14-08982643221086586] Gutiérrez N. C. (2014). Mexico: Availability and cost of health care–legal aspects. The Law Library of Congress. https://www.justice.gov/sites/default/files/eoir/legacy/2014/07/14/2014-010632%20MX%20RPT%20FINAL.pdf

[bibr15-08982643221086586] Hernández-Palacios R. D. Ramírez-Amador V. Jarillo-Soto E. C. Irigoyen-Camacho M. E. Mendoza-Núñez V. M. (2015). Relationship between gender, income and education and self-perceived oral health among elderly Mexicans. An exploratory study. Ciência & Saúde Coletiva, 20(4), 997–1004. 10.1590/1413-81232015204.00702014 25923612

[bibr16-08982643221086586] Jann B. (2008). The Blinder–Oaxaca decomposition for linear regression models. The Stata Journal, 8(4), 453–479. 10.1177/1536867X0800800401

[bibr17-08982643221086586] Kassebaum N. J. Smith A. G. C. Bernabé E. Fleming T. D. Reynolds A. E. Vos T. Murray C. J. L. Marcenes W. (2017). Global, regional, and national prevalence, incidence, and disability-adjusted life years for oral conditions for 195 countries, 1990–2015: A systematic analysis for the global burden of diseases, injuries, and risk factors. Journal of Dental Research, 96(4), 380–387. 10.1177/0022034517693566 28792274 PMC5912207

[bibr18-08982643221086586] Long J. S. Pavalko E. K. (2004). The life course of activity limitations: Exploring indicators of functional limitations over time. Journal of Aging and Health, 16(4), 490–516. 10.1177/0898264304265776 15271267

[bibr19-08982643221086586] Luo H. Bell R. A. Wright W. Wu Q. Wu B. (2018). Trends in annual dental visits among US dentate adults with and without self-reported diabetes and prediabetes, 2004-2014. The Journal of the American Dental Association, 149(6), 460–469. 10.1016/j.adaj.2018.01.008 29615188

[bibr20-08982643221086586] Malmö University & World Health Organization . (n.d.). Mexico—Oral health country/area profile project. Retrieved January 13, 2022, from https://capp.mau.se/country-areas/mexico/

[bibr21-08982643221086586] Marcenes W. Kassebaum N. J. Bernabé E. Flaxman A. Naghavi M. Lopez A. Murray C. J. L. (2013). Global burden of oral conditions in 1990-2010: A systematic analysis. Journal of Dental Research, 92(7), 592–597. 10.1177/0022034513490168 23720570 PMC4484374

[bibr22-08982643221086586] Marchini L. Reynolds J. C. Caplan D. J. Sasser S. Russell C. (2020). Predictors of having a dentist among older adults in Iowa. Community Dentistry and Oral Epidemiology, 48(3), 240–247. 10.1111/cdoe.12521 32043281

[bibr23-08982643221086586] Naka O. Anastassiadou V. Pissiotis A. (2014). Association between functional tooth units and chewing ability in older adults: A systematic review. Gerodontology, 31(3), 166–177. 10.1111/ger.12016 23170948

[bibr24-08982643221086586] Okoro C. A. Strine T. W. Eke P. I. Dhingra S. S. Balluz L. S. (2012). The association between depression and anxiety and use of oral health services and tooth loss. Community Dentistry and Oral Epidemiology, 40(2), 134–144. 10.1111/j.1600-0528.2011.00637.x 21883356

[bibr25-08982643221086586] Ortíz-Barrios L. B. Granados-García V. Cruz-Hervert P. Moreno-Tamayo K. Heredia-Ponce E. Sánchez-García S. (2019). The impact of poor oral health on the oral health-related quality of life (OHRQoL) in older adults: The oral health status through a latent class analysis. BMC Oral Health, 19(1), 141. 10.1186/s12903-019-0840-3 31291933 PMC6622000

[bibr26-08982643221086586] Parker SW Saenz J Wong R (2018). Health insurance and the aging: Evidence from the Seguro popular program in Mexico. Demography, 55(1), 361–386. 10.1007/s13524-017-0645-4 29357097 PMC5829015

[bibr27-08982643221086586] Peres M. A. Macpherson L. M. D. Weyant R. J. Daly B. Venturelli R. Mathur M. R. Listl S. Celeste R. K. Guarnizo-Herreño C. C. Kearns C. Benzian H. Allison P. Watt R. G. (2019). Oral diseases: A global public health challenge. Lancet, 394(10194), 249–260. 10.1016/S0140-6736(19)31146-8 31327369

[bibr28-08982643221086586] Ramírez M. Ahluwalia K. P. Teresi J. A. (2011). Correlates of dental visits among community-residing Latino elders: A public health alert. Gerodontology, 28(1), 12–18. 10.1111/j.1741-2358.2009.00335.x 19689744

[bibr29-08982643221086586] Salinas J. J. Al Snih S. Markides K. Ray L. A. Angel R. J. (2010). The rural – urban divide: Health services utilization among older Mexicans in Mexico. The Journal of Rural Health : Official Journal of the American Rural Health Association and the National Rural Health Care Association, 26(4), 333–341. 10.1111/j.1748-0361.2010.00297.x 21029168 PMC2967463

[bibr30-08982643221086586] Sánchez-García S. de la Fuente-Hernández J. Juárez-Cedillo T. Mendoza J. M. O. Reyes-Morales H. Solórzano-Santos F. García-Peña C. (2007). Oral health service utilization by elderly beneficiaries of the Mexican institute of social security in México city. BMC Health Services Research, 7(1), 211. 10.1186/1472-6963-7-211 18154658 PMC2245816

[bibr31-08982643221086586] Sánchez-García S. Heredia-Ponce E. Cruz-Hervert P. Juárez-Cedillo T. Cárdenas-Bahena Á. García-Peña C. (2014). Oral health status in older adults with social security in Mexico City: Latent class analysis. Journal of Clinical and Experimental Dentistry, 6(1), e29–e35. 10.4317/jced.51224 24596632 PMC3935902

[bibr32-08982643221086586] Spinler K. Aarabi G. Valdez R. Kofahl C. Heydecke G. König H.-H. Hajek A. (2019). Prevalence and determinants of dental visits among older adults: Findings of a nationally representative longitudinal study. BMC Health Services Research, 19(1), 590. 10.1186/s12913-019-4427-0 31429740 PMC6702718

[bibr33-08982643221086586] The World Bank (2018). Rural population (% of total population)-Mexico. The World Bank-Data. https://data.worldbank.org/indicator/SP.RUR.TOTL.ZS?locations=MX

[bibr34-08982643221086586] Torres J. M. Wong R. (2013). Childhood poverty and depressive symptoms for older adults in Mexico: A life-course analysis. Journal of Cross-Cultural Gerontology, 28(3), 317–337. 10.1007/s10823-013-9198-1 23783887 PMC3860586

[bibr35-08982643221086586] Wong R. Espinoza M. Palloni A. (2007). Adultos mayores Mexicanos en contexto socioeconómico amplio: Salud y envejecimiento. Salud pública de México, 49(S4), S436–S447. 10.1590/S0036-36342007001000002 17724516

[bibr36-08982643221086586] Wong R. Michaels-Obregon A. Palloni A. (2017). Cohort profile: The Mexican Health and Aging Study (MHAS). International Journal of Epidemiology, 46(2), e2–e2. 10.1093/ije/dyu263 25626437 PMC5837398

[bibr37-08982643221086586] Wong R. Ofstedal M. B. Yount K. Agree E. M. (2008). Unhealthy lifestyles among older adults: Exploring transitions in Mexico and the US. European Journal of Ageing, 5(4), 311–326. 10.1007/s10433-008-0098-0 25419206 PMC4239542

